# Perinatal Socioeconomic Disadvantage and Cardiovascular Comorbidities: National and State-Level Results of a Novel Cardio-Obstetrics Program

**DOI:** 10.3390/jcdd12080307

**Published:** 2025-08-13

**Authors:** Sakshi Sehgal, Elinita Pollard, Toscha Charles, Marquetta Thomas, Marlo Vernon, Gyanendra Sharma, Chadburn Ray

**Affiliations:** 1 Medical College of Georgia, Augusta University, 1120 15th St, Augusta, GA 30912, USA; 2 Georgia Prevention Institute, Augusta University, 1499 Walton Way, Augusta, GA 30901, USA; elpollard@augusta.edu (E.P.); mvernon@augusta.edu (M.V.); 3 Center for Health Equity Transformation, University of Kentucky, 760 Press Ave, Lexington, KY 40508, USA; 4 Division of Cardiology, Department of Medicine, Medical College of Georgia, Augusta University, 1120 15th St, Augusta, GA 30912, USA; tcharles@augusta.edu (T.C.); mathomas2@augusta.edu (M.T.); gsharma@augusta.edu (G.S.); 5 Department of Obstetrics and Gynecology, Medical College of Georgia, Augusta University, 1120 15th St, Augusta, GA 30912, USA; chray@augusta.edu

**Keywords:** cardiovascular risk, pregnancy, area deprivation index, socioeconomic disadvantage, social determinants of health

## Abstract

Perinatal cardiovascular disease accounts for nearly one-third of pregnancy-related deaths, with nearly 70% of these deaths preventable with appropriate cardio-obstetric care. The objective is to assess whether socioeconomic disadvantage, utilizing area deprivation index national and state rankings as a proxy, contributes to a higher risk of CV disease during the perinatal period. A single-site retrospective cohort study of 388 electronic health records patients seen at a novel cardio-obstetrics program between June 2022 and May 2024 was conducted. The main exposure was ADI state rankings, and the primary outcome of interest was diagnosis of perinatal CV disease, with secondary measures including preeclampsia, hypertension, and peripartum cardiomyopathy. Multivariable logistic regression models were utilized to examine the association between ADI and perinatal CV disease. National ADI ranking was associated with an increased odds of developing preeclampsia (OR: 2.56, 95% CI: 1.12–5.89) and HTN (OR: 2.37, 95% CI: 1.19–4.72). Socioeconomic disadvantage during the perinatal period is associated with a statistically significant risk of CV disease, including preeclampsia, hypertension, and peripartum cardiomyopathy, as well as any CV diagnosis in general.

## 1. Introduction

In the United States, cardiovascular (CV) disease is the leading cause of perinatal mortality, with morbidity and associated effects only increasing in the past several decades [1]. Hypertensive disorders, including hypertension and preeclampsia, comprise the most commonly encountered CV disease in pregnancy and in the perinatal period [2]. They contribute to dangerous health outcomes such as congestive heart failure, pulmonary edema, and mortality [2,3]. Pregnant patients in Georgia (GA) may experience CV disease at a disproportionate rate, as maternal mortality rates in GA exceed the national average [4]. Given the pervasiveness of hypertensive diseases during pregnancy and the excessive mortality rate in GA, it is imperative to examine the association between hypertensive disorders during pregnancy and factors beyond the individual level.

Social determinants of health (SDOH) can further complicate this interplay of underlying pathophysiologic factors. When considering aggregated SDOH, there is worsened CV disease burden in pregnant patients with an increased burden of socioeconomic factors ranging from neighborhood deprivation to limited access to transportation [5]. There is evidence to suggest that neighborhood-level factors can exacerbate underlying pathophysiology or contribute to novel presentations of disease. The area deprivation index (ADI), a composite measure of neighborhood disadvantage, has been linked to adverse pregnancy outcomes [6,7]. For example, Lemon and colleagues found living pregnant patients residing in the most disadvantaged neighborhoods were 1.37 times more likely to have stage 2 hypertension at 3 weeks postpartum than those living in the least disadvantaged neighborhoods [7]. Despite previous studies examining the relationship between neighborhood deprivation and hypertensive outcomes during pregnancy, the association between neighborhood disadvantage and perinatal disease remains unexamined in the context of an active intervention [7,8,9].

While prior studies have identified a link between ADI and CV outcomes in pregnancy, none to our knowledge have specifically looked at both state- and national-level ADI rankings and subsequent CV effects in the context of a novel cardio-obstetrics program. Thus, this study sought to assess the association between socioeconomic disadvantage, defined using ADI and self-identified patient characteristics, in the perinatal period with incidence of CV disease (i.e., preeclampsia, hypertension, and peripartum cardiomyopathy) [2]. We hypothesized that greater socioeconomic disadvantage, measured based on lower state and national ADI rankings, would be significantly associated with perinatal CV disease. Evaluation of neighborhood areas of deprivation using the ADI can increase understanding of salient measures of long-term perinatal outcomes, within and beyond the clinical setting.

## 2. Materials and Methods

### 2.1. Study Design

Between June 2022 and May 2024, 707 patients were seen in a novel cardio-obstetrics program at a single site in Augusta, Georgia. The program enrolled participants who were identified and/or referred due to underlying CV comorbidity or new diagnosis of CV disease in the perinatal period. Study analysis utilized national and state area deprivation index (ADI) rankings and patient-identified characteristics as the exposure variable. The primary outcome measure was CV diagnosis, with a secondary focus on specific CV diagnoses of preeclampsia, hypertension, and peripartum cardiomyopathy (PPCM). This study was approved by the Augusta University Institutional Review Board (IRB Approval #2087603-1; Approval Date: 4 October 2023).

### 2.2. Study Population

Patients over 18 years of age identified and/or referred as high-risk for CV disease or patients with a new diagnosis of CV disease in the perinatal period referred to the single-site cardio-obstetrics program between June 2022 and May 2024 were selected for this study. Study participants included patients with at least one scheduled visit at the cardio-obstetric clinic, which included self-identification of patient demographic characteristics and tracking of CV diagnoses and any subsequent complications during the perinatal period from the initial prenatal visit through delivery. Patients with medical record numbers (MRNs), recorded 9-digit home address zip code, and residence in the states of Georgia and South Carolina were included. Patients with insufficient demographic characteristic responses, states of residence outside Georgia or South Carolina (due to insufficient study participants from other states), and unavailable ADI state or national rankings were excluded from analysis. Finally, duplicate and follow-up visits for individual patients attending the cardio-obstetrics program were excluded from analysis.

### 2.3. Exposure

The primary exposure variable was neighborhood socioeconomic disadvantage measured using national and state ADI rankings. During the first intake visit at the cardio-obstetrics program, the patient’s MRN and geocoded 9-digit home address zip code were recorded. The specific zip codes were used to link each de-identified patient with the corresponding 2021 ADI national and state ranking values, created using 2016–2020 American Community Survey national and state data. Utilizing the 2021 version of the ADI allowed for the most current analysis using updated census block data.

ADI describes a measure of community socioeconomic deprivation utilizing 17 census variables, such as median family income, income disparity, families below the poverty level, and population below 150% of the poverty level. Provided by the University of Wisconsin’s Center for Health Disparities Research, the ADI provides aggregate data and ranking of neighborhood deprivation based on census block groups, ranging from 1 to 100, with 100 describing neighborhoods with the greatest socioeconomic deprivation [10,11].

ADI data are obtained from the American Community Survey and the University of Wisconsin Center for Health Disparities Research [10,11]. For this analysis, ADI values were dichotomized into quartiles for national ranking values and into quintiles for state ranking values.

### 2.4. Covariates

Covariates included patient-identified demographic characteristics. At the first intake visit to the cardio-obstetrics program, participants could opt to self-identify race, access to transportation, and additional children. Study analysis dichotomized race into Black, White, and Hispanic/Other. Patients self-identifying as South Asian, multiple races, or unspecified were categorized as “Other” due to insufficient cross-sectional sample size. Multiparity was dichotomized into yes, no, or unknown categories.

### 2.5. Outcomes

The primary outcome was diagnosis of CV disease in the perinatal period. Initial patient visits following referral or enrollment in the cardio-obstetrics program detailed initial CV diagnoses preceding referral. Follow-up visits detailed additional CV diagnoses or complications during the perinatal period. The study analysis solely included final CV diagnoses. Secondary outcome measures specified 3 CV diagnoses: (1) preeclampsia, (2) hypertension, and (3) peripartum cardiomyopathy. To prioritize sample size, at least 1 encounter with a secondary outcome diagnosis was sufficient for inclusion within that sub-category. Furthermore, presence of at least 1 additional CV comorbidity beyond the primary CV diagnosis during the perinatal period/follow-up with the cardio-obstetrics program sufficed for inclusion within that analysis. We did not specify the source of referral.

### 2.6. Statistical Analysis

To better understand the role of additional socioeconomic risk factors on CV disease in pregnancy, we used descriptive statistics for the demographic variables of race and multiparity. We conducted chi-square tests to compare categorical variables to identify bivariate differences in perinatal CV diagnoses based on sociodemographic patient characteristics of race and number of additional children.

Statistical analysis was performed between December 2023 and June 2024. We used multivariable logistic regression to evaluate the association between the exposure variable of ADI ranking (state quintile and national quartile) and outcome measure of CV diagnosis in the perinatal period. We calculated the regression coefficient with 95% confidence intervals.

Models were adjusted based on sociodemographic covariates of patient-reported race and additional children. We did not adjust for other reported characteristics due to insufficient sample size at the intake visit and/or minimal effect on the possible association between socioeconomic disadvantage/neighborhood deprivation and CV disease in the perinatal period. All the statistical analyses were performed using the SAS version 9.4M8 (SAS Institute, Cary, NC, USA) and SPSS statistical software, version 29.0.2.0 (IBM, Armonk, NY, USA).

## 3. Results

Of the 707 recorded visits at the cardio-obstetrics program between June 2022 and May 2024, 472 (66.7%) participants met the inclusion criteria following duplicate/follow-up removal and excluding patients without identifiable zip codes or provided demographic information. A total of 84 participants were removed due to absent ADI rankings given inadequate population or high group quarters, as well as non-residents of Georgia or South Carolina. A total of 388 participants were included in the final analysis.

As shown in Table 1, most patients lived in the most disadvantaged state ADI quintile (42.8%) and national ADI quartile (64.2%); did not have PPCM (91.8%), preeclampsia (71.9%), or HTN (52.1%); and only had one diagnosis at most (79.6%). Of note, the increased percentage representation of PPCM patients relative to the general population can be attributed to increased rates of referral to the novel program. The patients most frequently did not have a completed echocardiogram (47.4%), were 25–34 years old (51.5%), Black (52.6%), were seen from 2023 to 2024 (60.1%), and had at least one child (85.1%). In total, 20.4% of the patients had two to three CV diagnoses during the perinatal period. A total of 60.1% of the patient cohort was seen in 2023–2024, and 39.9% was seen in 2022–2023. We observed significant differences in developing preeclampsia based on the state (*p* = 0.003) and national (*p* = 0.006) ADI rankings. The same was observed for hypertension (state: *p* = 0.039; national: *p* = 0.010) and whether the patients had more than one of these conditions (state: *p* = 0.034; national: *p* = 0.039).

In unadjusted models, there was a significant association between the state ADI ranking and HTN. Compared to those living in the first quintile, the patients living in the fourth quintile had an increased odds of HTN (OR: 3.21, 95% CI: 1.14–9.08). However, this association was no longer significant after adjustment (Table 2).

The national ADI ranking was significantly positively associated with CV conditions in both the adjusted and unadjusted models, as shown in Table 3. The patients living in the fourth ADI quartile had 2.56 increased odds of developing preeclampsia (OR: 2.56, 95% CI: 1.12–5.89) and 2.37 increased odds of HTN (OR: 2.37, 95% CI: 1.19–4.72).

## 4. Discussion

Neighborhood socioeconomic disadvantage, measured using the ADI national and state rankings, was significantly associated with increased risk of CV disease, specifically preeclampsia and hypertension, in the perinatal period in a large, diverse patient cohort over two years. These results underscore the interplay between social determinants of health and socioeconomic risk factors and CV disease in pregnancy and the perinatal period [12,13].

This study highlights the association between the underlying neighborhood environmental factors and CV disease in the perinatal period. There is an increased risk of both CV disease generally, as well as specific CV diagnoses of preeclampsia and HTN, attributed to socioeconomic disadvantage and neighborhood deprivation quantified using ADI rankings [5,14,15]. Prior research describes the role of socioeconomic risk factors such as decreased neighborhood income in a higher risk of CV disease and risk for both the mother and child [16]. Disadvantageous social and economic conditions such as increased rates of violence can further contribute to the greater risk of CV disease [17,18]. Effects of these conditions are often amplified in pregnancy and in the perinatal period, coupled with any incidence of underlying disease such as diabetes or hypertension [19,20].

This study evaluated the association between worse neighborhood deprivation/socioeconomic circumstances with CV disease in the perinatal period. Incidence of CV disease was the primary outcome measure, with secondary outcome analysis including specific CV diseases of preeclampsia, hypertension, and peripartum cardiomyopathy. Analysis in both the crude and adjusted models revealed increased odds of preeclampsia and hypertension in the lowest quartile and quintile for national and state models, respectively.

The study results raise several points for further speculation. Increased risk of hypertension was associated with the fourth quintile ADI in unadjusted models; however, this was not the case for the fifth quintile. For the adjusted models, variation in factors such as multiparity could have decreased statistical power, thus leading to non-significant findings. Similarly, statistical significance was not demonstrated for PPCM diagnosis and association with national or state ADI. This could be due to the reduced sample size of patients (n = 32) with PPCM diagnosis relative to other diagnoses. Additionally, increased rates of referral for PPCM could also contribute to variation relative to the general study population.

Confounding patient-specific factors and additional diagnoses within the small sample size could further affect the association. Outcome variation between the national and state ADI may be driven by percentile versus decile specificity in the calculation of the index itself. Aggregated trends in data and broader generalizability may contribute to higher statistical power and subsequent statistical significance in models considering national versus state ADI.

These findings demonstrate the critical interplay between social determinants of health and CV outcomes in the perinatal period [21]. Increased risk of CV disease in lower-ranked ADI quartiles and quintiles underscores the importance of novel diagnostic and therapeutic interventions such as the cardio-obstetrics program that afford routine follow-up care for patients, along with large-scale community interventions. Early, routine intervention and follow-up, along with community-scale guidelines, can help decrease environment-associated risk and promote maternal CV health [22,23]. This study further affirms evidence that socioeconomic risk factors can significantly affect maternal health, including increasing likelihood of short- and long-term maternal cardiac health complications. Evaluating this relationship in the context of a diverse cohort affected by various CV diagnoses in a single-site study further underscores the importance of preventive care and interdisciplinary management.

### 4.1. Clinical and Research Implications

The findings of this study provide a better understanding of the underlying socioeconomic and environment-based risk factors that can contribute to increased incidence of CV disease in pregnancy and the perinatal period. Several implications exist from a clinical perspective. Firstly, clinicians can utilize patient-identified challenges in seeking care, prior CV history, or any history of CV-related comorbidities to accelerate seeking referrals and interdisciplinary care between cardiology and obstetrics. Secondly, routine follow-up care in the interdisciplinary care setting further permits dynamic evaluation of CV diagnoses and complications while incorporating earlier therapeutic intervention and patient-tailored care in the prenatal, perinatal, and postpartum periods [22,23]. Thirdly, a better understanding of socioeconomic risk factors can allow clinicians to identify high-risk patients earlier and provide care for subsequent pregnancies, as well [24].

Bridging interdisciplinary care with community-specific support services and resources provides additional opportunity [19,25]. Cardio-obstetrics programs often integrate social workers, nutritional and diet resources, and additional services that can help reduce challenges with accessing care, such as transportation and scheduling routine follow-up visits [22,23].

This study sought to investigate the relationship between neighborhood socioeconomic disadvantage and perinatal CV disease risk. Additional research is needed to better understand the role of specific environments and socioeconomic factors in development of CV disease [18,25]. Specifically, further studies are necessary to understand the role of each individual social determinant, such as presence of neighborhood violence, income below the poverty line, and patient risk factors such as prior medical and surgical history, in contributing to CV disease incidence in the perinatal period. Additional data from similar cohort studies at cardio-obstetrics programs could also permit comparison between rates of risk of CV disease based on ADI rankings and patient characteristics [23]. Furthermore, the differences in the results attributed to the state versus national ADI can also have implications. For instance, the state ADI may be less nuanced than the national ADI in reporting subtle variations with far-reaching implications in CV health outcomes. Further research could also work to better understand the role of neighborhood deprivation in shaping rates of diagnoses such as tachycardia or hypertrophic obstructive cardiomyopathy with small patient sample sizes [26,27]. Another avenue of research could utilize baseline data from this study’s findings to longitudinally compare rates of CV disease before and after interdisciplinary diagnostic and therapeutic intervention through cardio-obstetrics programs. Analysis of effects of early referral and intervention could further tailor when and how such programs are implemented within patient care.

### 4.2. Strengths and Limitations

This study utilized data from a diverse cohort of patients at a large academic medical center across two years, including patients from various geographic, racial, and socioeconomic backgrounds, promoting generalizability of the study findings. Additional strengths include standardization of data and use of instruments widely accessible for the primary exposure variable. Consideration of both state and national level ADI rankings further permits understanding of neighborhood deprivation at two separate levels of individual census block groups.

Limitations of the study findings include that CV diagnosis was assessed based on the final follow-up visit for each patient. As a result, patients with earlier diagnoses that were later resolved by a later visit may not be as accurately reflected in the final assessment of CV diagnoses. Secondly, patient-identified characteristics were used to assess association based on race and number of additional children, but additional sociodemographic characteristics, such as level of education and household income, were missing. Thirdly, adjusted models did not account for prior patient history of CV disease, including current medications or prior surgeries/interventions [28]. Furthermore, the secondary outcome measure of hypertension included diagnoses both prior to and during the gestational period, including both chronic and gestational hypertension diagnoses, along with diagnoses for which the time period was not specified [29]. This study was additionally limited to two states—Georgia and South Carolina—due to sample size based on patient home address zip code. In order to further address these limitations, we suggest several key avenues for future research, including expanding the study sample to additional geographic regions and states to further validate study findings, integrating longitudinal patient data, and adjusting for additional comorbidities and patient-specific factors.

## 5. Conclusions

Socioeconomic disadvantage and neighborhood-level deprivation are associated with increased risk of CV disease, including preeclampsia and hypertension, in the perinatal period. The results of this study emphasize the importance of considering social determinants of health in assessing patient risk for CV disease. Patient-specific interdisciplinary interventions, such as cardio-obstetrics programs and identification of high-risk patients, can further help address increased CV risk in the perinatal period.

## Figures and Tables

**Table 1:** Sample Characteristics.

	Total (n = 388)	PPCM		Preeclampsia		Hypertension	
		Yes (n = 32, 8.2%)	No (n = 356, 91.8%)	Yes (n = 109, 28.1%)	No (n = 279, 71.8%)	Yes (n = 186, 47.9%)	No (n = 202, 52.1%)
**State ADI quintile**							
First	21 (5.4%)	1 (3.1%)	20 (5.6%)	4 (3.7%) **	17 (6.1%) **	6 (3.2%) *	15 (7.4%) *
Second	52 (13.4%)	7 (21.9%)	45 (12.6%)	8 (7.3%) **	44 (15.8%) **	19 (10.2%) *	33 (16.3%) *
Third	53 (13.7%)	1 (3.1%)	52 (14.6%)	9 (8.3%) **	44 (15.8%) **	22 (11.8%) *	31 (15.3%) *
Fourth	96 (24.7%)	8 (25.0%)	88 (24.7%)	39 (35.8%) **	57 (20.4%) **	54 (29.0%) *	42 (20.8%) *
Fifth	166 (42.8%)	15 (46.9%)	151 (42.4%)	49 (45.0%) **	117 (41.9%) **	85 (45.7%) *	81 (40.1%) *
**National ADI quartile**							
First and second	49 (12.6%)	6 (18.8%)	43 (12.1%)	7 (6.4%) **	42 (15.1%) **	15 (8.1%) **	34 (16.8%) **
Third	90 (23.2%)	5 (15.6%)	85 (23.9%)	19 (17.4%) **	71 (25.4%) **	39 (21.0%) **	51 (25.2%) **
Fourth	249 (64.2%)	21 (65.6%)	228 (64.0%)	83 (76.1%) **	166 (59.5%) **	132 (71.0%) **	117 (57.9%) **
**Age**							
20–24	75 (19.3%)	2 (6.3%) ***	73 (20.5%) ***	23 (21.1%)	52 (18.6%)	29 (15.6%) *	46 (22.8%) *
25–34	200 (51.5%)	10 (31.3%) ***	190 (53.4%) ***	49 (45.0%)	151 (54.1%)	93 (50.0%) *	107 (52.9%) *
35–44	113 (29.1%)	20 (62.5%) ***	93 (26.1%) ***	37 (33.9%)	76 (27.2%)	64 (34.4%) *	49 (24.3%) *
**Race**							
Black	204 (52.6%)	23 (71.9%) *	181 (50.8%) *	26 (23.9%) *	110 (39.4%) *	56 (30.1%) **	80 (39.6%) **
White	136 (35.1%)	5 (15.6%) *	131 (36.8%) *	68 (62.4%) *	136 (48.7%) *	114 (61.3%) **	90 (44.6%) **
Other	48 (12.4%)	4 (12.5%) *	44 (12.4%) *	15 (13.8%) *	33 (11.8%) *	16 (8.6%) **	32 (15.8%) **
**Children**							
No	58 (14.9%)	1 (3.1%)	57 (16.0%)	10 (9.2%) *	48 (17.2%) *		
Yes	330 (85.1%)	31 (96.9%)	299 (84.0%)	99 (90.8%) *	231 (83.8%) *		
**Echocardiogram**							
No data	184 (47.4%)	21 (65.6%)	163 (45.8%)	56 (51.4%)	128 (45.9%)	95 (51.1%)	89 (44.1%)
Ordered	147 (37.9%)	9 (28.1%)	138 (38.8%)	41 (37.6%)	106 (38.0%)	72 (38.7%)	75 (37.1%)
Completed	57 (14.7%)	2 (6.3%)	55 (15.4%)	12 (11.0%)	45 (16.1%)	19 (10.2%)	38 (18.8%)
**Year seen**							
2022–2023	155 (39.9%)	14 (43.8%)	141 (39.6%)	30 (27.5%) **	125 (44.8%) **	66 (35.5%)	89 (44.1%)
2023–2024	233 (60.1%)	18 (56.3%)	215 (60.4%)	79 (72.5%) **	154 (55.2%) **	120 (64.5%)	113 (55.9%)

Abbreviations: ADI, area deprivation index; PPCM, peripartum cardiomyopathy. Bold text indicates statistical significance. * *p* < 0.05, ** *p* < 0.01, *** *p* < 0.001.

**Table 2:** Association between state area deprivation index quartile and cardiovascular outcomes.

	PPCM		Preeclampsia		Hypertension	
	Unadjusted	Adjusted ^a^	Unadjusted	Adjusted ^a^	Unadjusted	Adjusted ^a^
**State ADI Quintile**						
First	Reference	Reference	Reference	Reference	Reference	Reference
Second	3.11 (0.35–27.56)	2.23 (0.24–20.70)	0.77 (0.20–2.94)	0.60 (0.16–2.19)	1.44 (0.47–4.38)	1.19 (0.38–3.73)
Third	0.39 (0.02–6.63)	0.18 (0.10–3.19)	0.87 (0.23–3.24)	0.69 (0.20–2.43)	1.77 (0.59–5.35)	1.29 (0.42–4.00)
Fourth	1.81 (0.21–15.69)	1.23 (0.14–11.2)	2.91 (0.90–9.41)	2.25 (0.74–6.87)	**3.21 (1.14–9.08)**	2.58 (0.89–7.48)
Fifth	1.99 (0.24–16.18)	1.17 (0.13–10.23)	1.78 (0.56–5.62)	1.27 (0.42–3.82)	2.62 (0.96–7.15)	2.10 (0.74–5.95)

Abbreviations: ADI: area deprivation index; PPCM, peripartum cardiomyopathy. ^a^ Models adjusted for age, race, kids, echocardiogram, and year the patient was seen. Bold text indicates statistical significance.

**Table 3:** Association between national area deprivation index quartile and cardiovascular outcomes.

	PPCM		Hypertension		Preeclampsia	
	Unadjusted	Adjusted ^a^	Unadjusted	Adjusted ^a^	Unadjusted	Adjusted ^a^
**National ADI Quartile**						
First and second	Reference	Reference	Reference	Reference	Reference	Reference
Third	0.42 (0.12–1.47)	0.28 (0.08–1.01)	1.73 (0.83–3.63)	1.52 (0.71–2.91)	1.61 (0.62–4.17)	1.48 (0.58–3.77)
Fourth	0.66 (0.25–1.74)	0.47 (0.18–1.24)	**2.56 (1.32–4.95)**	**2.37 (1.19–4.72)**	**3.00 (1.28–7.01)**	**2.56 (1.12–5.89)**

Abbreviations: ADI, area deprivation index; PPCM, peripartum cardiomyopathy. ^a^ Models adjusted for age, race, kids, echocardiogram, and year the patient was seen. Bold text indicates statistical significance.

## Data Availability

The original contributions presented in this study are included in the article. Further inquiries can be directed to the corresponding author.

## Abbreviations

The following abbreviations are used in this manuscript:

CV	Cardiovascular
GA	Georgia
SDOH	Social determinants of health
PPCM	Peripartum cardiomyopathy
ADI	Area Deprivation Index
MRN	Medical record number
